# Neurasthenia

**Published:** 1900-09

**Authors:** Virginius W. Gayle

**Affiliations:** Professor of Materia Medica and Therapeutics, University Medical College, Kansas City, Missouri


### Neurasthenia.

VIRGINIUS W. GAYLE, M. D.,
Professor of Materia Medica and Therapeutics, University Medical College,
Kansas City, Missouri.

Neurasthenia is a veritable "Pandora's Box." I venture to say that a patient suffering from this condition can have any symptom desired; can have a pain in any portion of the economy, and can have any disease in the category, unless, as Jerome says: "A man can not have 'housemaid's knee.'" And I further aver that it is not all "imaginitis" either. The rapidity with which symptoms shift and change is something remarkable. Tachycardia, bradycardia, and arrythenia are common, pain at McBurney's point, lancinating pains in the limbs resembling locomotor ataxia, fear of impending paralysis, symptoms of diabetes or Bright's disease, are frequently seen.

Neurasthenia seems to be the coming American disease. If the nervous system does an increased amount of work, nutritive material is not brought to the centers of activity in sufficient quantity, and the waste products are not carried away as rapidly as is necessary. On the one hand there is malnutrition, and, on the other hand, absorption of toxic materials which produces the multiform nervous manifestations.

There are usually a neurotic stigmata, manifested by cephalalgia, cerebral depression, general weakness, loss of motor powers, dyspepsia, and in many cases insomnia; but insomnia is not met with in the majority of cases. Often there is a condition of sleepiness, which manifests itself at any or all times. The patient feels sleepy the greater portion of the time. Cold hands and feet, hyperidrosis, hypochondria and hyperesthesia are common symptoms. Where spinal symptoms predominate, physical exercise is contraindicated. I am of the opinion that the digestive apparatus is one of the prime factors in the cause of the disturbances. The pupils are sometimes habitually dilated, and there are changes in the optic nerves. Dilatation of the stomach is a frequent concomitant; numbness and tingling are also disturbing symptoms. There is a neurasthenic diathesis beyond question.

Neurasthenia is pathological fatigue. As the toxic products of fatigue are always found in nerve and muscular tissues and may accumulate in the blood, they contribute to auto-intoxication, deficient energy and inanition, with loss of mental power, worry, irritability, hyperesthesia, langour and anaesthesia. As some one has aptly called it, "morning tire" is nearly always present. Many cases are called "rheumatics." They suffer from transitory pains in all muscular regions, pleurodynia in an aggravated form, pain radiating to the axillary space, pains in the limbs, possibly ataxic symptoms, gastro-intestinal and sexual disturbances. Heterrophoria is certainly a potent factor in the production of neurasthenia. Many of the cases have eye-strain. I know of one case in which there was severe pain, referable to the middle of the hemispheres, when the right eye was turned outward. Tenotomy for esophoria gave instant relief to this symptom. Sometimes graduated tenotomies or Steven's advancement operation may be done. I have had a number of cases of neurasthenia markedly benefited, and some cases cured, by having my oculist relieve the eye-strain by the necessary procedure.

Splanchnoptosis or gastrectasia may be causes of the disease. The ptosis of any viscus may produce the trouble. Glenard's enteróptosis, Bouchard's gastrectasia, or Beard's nervous theory may all be etiological factors. The dyspepsia symptoms are varied. There may be super-acidity, or, conversely, sub-acidity, or an entire absence of acid. There may be hypersecretion. Fermentation is frequent with an accumulation of enormous quantities of gas; eructations that are distressing, thoracic pains of great intensity, all depend upon the gas in the stomach. When this is gotten rid of, the patient is fairly comfortable. The trouble generally is the socalled "amylaceous dyspepsia"; the starches are not digested. Therefore let your patient eschew starches, particularly potatoes, hot white bread, pastry, etc. Corn bread may be used and moderate quantities of wheat bread, if stale.

Neurasthenia and hysteria are entirely different. Neurasthenics usually "go over the bridge before they get to it." Lithemia is generally present; a better word, perhaps, would be "uricacidemia." There is invariably present myasthenia. Arterio-sclerosis is found in many cases. The special senses are frequently disturbed, particularly vision. There is a sense of weariness or aching of the eyeballs, constant desire to close the lids—a condition of pseudoptosis of the lids altogether disagreeable. The auditory apparatus is also frequently involved. Most patients have morbid doubts, with a feeling of great anxiety, sometimes leading to impulsive acts. These attacks of "blues" irresistibly force themselves upon the patient, and are often impossible to cast off. Some have a morbid fear of heights, bridges, etc. They usually magnify their symptoms and are always looking for some terrible calamity or disease to overtake them. Dr. Beard says: "The belief that all patients magnify their symptoms is erroneous. Many persons overlook symptoms or forget them. Such symptoms as sweating scrotum, palmar hyperidrosis, cold hands and feet, pain in the perineum, dryness of the hair and skin, flying pains in different parts of the body, are not regarded by these persons as evidences of disease."

There are individuals suffering from this disease, who are mental, moral or sexual perverts. They lie, steal, drink and abuse the sexual organs. Many cases of sexual neurasthenia in the male may be traced to phimosis or irritation of the prostate gland. There may be neurasthenic asthenopia, contraction of the field of vision, lessened acuteness of vision, imperfect images on the retina, and sometimes photophobia.

Whether neurasthenia and insanity are so closely allied as some think, is not proven. I believe the line of demarkation is more closely drawn than some teach. Neurasthenia may be summarized as a condition charcterized by weakness and irritability of the nervous functions, in greater or less degree, depending on the patient and the environment of the patient. All neurasthenics are not irritable or moody; some are bright and cheerful even when suffering from some of the nervous symptoms mentioned. Flashes of heat and hyperemia of the skin are often seen in women who have the disease at the menopause. Is it possible that a "change of life" takes place in the male? Why not? Many of the cases occur between the ages of forty-five and fifty. Cardiac symptoms are common, pain in the precardial region, a sense of suffocation. The slightest excitement increases the action of the heart to an abnormal degree. Symptoms of aneurism manifest themselves, sometimes in the thoracic and in other cases in the abdominal aorta. Tremor is very common, almost to the extent of seeming paralysis agitans. Suicidal impulses are sometimes met with in neurasthenics, but they are not nearly so common as in melancholia.

As to the pathology of neurasthenia, we are still in the dark, and know very little about it. There may be changes in the nerve cell itself. The physiological action of the nervous elements may be inhibited by the absorption of the toxines spoken of before. One of the chief symptoms is amyosthenia.

*Treatment.* Rest and change of environment are above everything else. A sea voyage to Australia will prove a great blessing to every neurasthenic who takes it. The "rest cure" is in some cases the *"sine qua non"*; in other cases that I have seen it does no good. Dietetics and massage enter largely into the treatment of this disease. Peptonized milk as an exclusive diet in some cases is of great value. A glass of sherry or claret is often beneficial, and sometimes beer can be taken with advantage. Somatose, which is an albuminous extract of meat and contains the nourishing elements in a readily digested form, is frequently of great benefit. It is taken up rapidly and in quantities to insure against the evils of malnutrition. It seems to have a happy effect in cases of sexual neurasthenia, and does not cause flatulence or diarrhea as so many foods do. In some cases of neurasthenia there is anemia with marked loss of hemoglobin. These cases require a chalybeate. Organic ferruginous preparations are much better tolerated than inorganic. The former scarcely ever produce gastric or intestinal irritation; particularly is this the case with ferro-somatose, which is a combination of albumoses with organic iron. In all cases of impaired digestion, weakness and emaciation, so often found in neurasthenics, this preparation relieves the condition, increases the number of red blood corpuscles, improves the appetite, and produces a gain in weight. It is an efficient nutritive and roborant, readily absorbed, and can be given without the knowledge of the patient. It has a good effect in nourishing and relieving the depressed condition of the nervous system. It may be given in warm milk, chocolate, or soup, three or four times a day, to the extent of 10 gm. pro die. This is equivalent to the iron in about ten Blaud's pills. Strychnine, arsenic and gold are indicated and often subserve a good purpose. The "trinity pill" in many cases allays the nervous irritation in a charming manner. Laxatives, when indicated, preferably the salines, and hypnotics are often needed. Solution of bromide of strontium will sometimes give relief and produce less gastric disturbances than the other members of this group. Trional in 15 grain doses, two hours before retiring, will, in most cases, produce sleep and afford relief from that *bete noir,* insomnia. It has no tendency to produce habituation and there is an absence of cumulative effects. In the insomnia of neurasthenia it produces sleep in many instances in which other remedies fail. It does not seem to depress the heart, and has no injurious action on the kidneys or digestive organs. All in all, it is the best remedy of its class. When muscular pains are distressing, as they often are, 10 grains of Salophen three times a day will give good results; being a combination of salicylic acid and Phenacetin, it is well seen how it can be of advantage. Salophen passes through the stomach unchanged, and does not disturb gastric digestion. It does not produce tinnitus aurium or cerebral pressure, and is indicated in all painful affections, particularly those of a neuralgic type.

